# Metabolic Dysfunction-Associated Steatotic Liver Disease in Inflammatory Bowel Disease: A Cross-Sectional Study of Prevalence and Associated Factors

**DOI:** 10.3390/metabo16070450

**Published:** 2026-06-26

**Authors:** Hüseyin Aykut, Nermin Mutlu Bilgiç, Duygu Demirtaş, Süleyman Sayar, Kamil Özdil

**Affiliations:** 1 Department of Gastroenterology, Umraniye Training and Research Hospital, 34764 Istanbul, Türkiye; drnerminmutlu@yahoo.com.tr; 2 Department of Radiology, Umraniye Training and Research Hospital, University of Health Sciences, 34764 Istanbul, Türkiye; drduygudemirtas@icloud.com; 3 Department of Gastroenterology, VM Medical Park Pendik Hospital, 34899 Istanbul, Türkiye; drssayar@gmail.com; 4 Department of Gastroenterology, Memorial Atasehir Hospital, 34634 Istanbul, Türkiye; kamilozdil@gmail.com

**Keywords:** inflammatory bowel disease, metabolic dysfunction-associated steatotic liver disease, Crohn’s disease, ulcerative colitis, metabolic risk factors, hepatic steatosis

## Abstract

**Highlights:**

**What are the main findings?**

- MASLD prevalence was high (61.3%) among selected patients with IBD in this Turkish tertiary-care cohort who had abdominal imaging available within the previous 6 months.
- Age, body mass index, and triglyceride levels were independently associated with MASLD, whereas disease-related IBD characteristics were not.

**What are the implications of the main findings?**

- MASLD in patients with IBD appears to be more closely associated with metabolic dysfunction than with IBD-related clinical characteristics and may largely reflect the underlying cardiometabolic profile of the background population.
- Routine metabolic risk assessment and proactive MASLD screening should be considered as part of long-term IBD management, particularly in populations with a high cardiometabolic burden.

**Abstract:**

**Background/Objectives**: Metabolic dysfunction-associated steatotic liver disease (MASLD) highlights the central role of metabolic dysregulation in hepatic steatosis. Patients with inflammatory bowel disease (IBD) are increasingly recognized to have an adverse cardiometabolic risk profile; however, data regarding MASLD prevalence and associated factors in this population remain limited, particularly in regions with a high metabolic burden such as Türkiye. This study aimed to determine the prevalence of MASLD in patients with IBD and to identify the metabolic and disease-related factors associated with its development. **Methods**: In this retrospective cross-sectional study, adult patients with IBD followed at a tertiary referral center in Türkiye were included. Hepatic steatosis was assessed using routine abdominal imaging, and MASLD was defined according to the 2023 Delphi Consensus criteria. Clinical, demographic, and metabolic variables were analyzed. Multivariable logistic regression analysis was performed to identify factors independently associated with MASLD. **Results**: A total of 194 IBD patients who had abdominal imaging available within the previous 6 months were included, of whom 61.3% had MASLD. MASLD was more prevalent in patients with UC than in those with CD (70.7% vs. 55.5%, *p* = 0.036). However, UC diagnosis was not independently associated with MASLD after multivariable adjustment. Patients with MASLD were older and had higher body mass index and less favorable metabolic profiles. In multivariable analysis, age (OR 1.055, 95% CI 1.022–1.089; *p* < 0.001), body mass index (OR 1.199, 95% CI 1.092–1.316; *p* < 0.001), and triglyceride levels (OR 1.012, 95% CI 1.004–1.020; *p* = 0.005) were independently associated with MASLD. In contrast, disease-related factors, including disease activity, biologic therapy, and prior surgery, were not independently associated with MASLD. **Conclusions**: MASLD prevalence was high among selected IBD patients with available abdominal imaging and appears to be more strongly associated with metabolic risk factors than with disease-specific characteristics. In populations with a high background cardiometabolic burden, MASLD in IBD may largely reflect the underlying regional metabolic milieu. These findings support the integration of metabolic risk assessment and proactive MASLD screening into routine IBD care. Prospective longitudinal studies are needed to clarify the causal relationship between metabolic dysfunction, IBD-related factors, and MASLD progression.

## 1. Introduction

Inflammatory bowel diseases (IBD), including ulcerative colitis (UC) and Crohn’s disease (CD), are chronic, relapsing inflammatory disorders with diverse extraintestinal manifestations (EIM). The global prevalence of IBD has been steadily increasing over recent decades, reflecting changes in environmental exposures and lifestyle factors, and is expected to become a substantial global health burden in the near future [1,2]. Beyond gastrointestinal involvement, patients with IBD are increasingly recognized to have an elevated cardiometabolic risk profile, including insulin resistance, dyslipidemia, and altered body composition [3]. These changes are thought to result from a complex interplay between chronic inflammation, altered gut permeability, disease-related factors, and medical therapies and may contribute to an increased long-term risk of cardiometabolic complications.

Given this metabolic burden, patients with IBD may be particularly susceptible to hepatic steatosis. Since June 2023, the term metabolic dysfunction-associated steatotic liver disease (MASLD) has replaced non-alcoholic fatty liver disease (NAFLD), formerly defined as hepatic steatosis in the absence of secondary causes such as alcohol consumption, to more accurately reflect the primary role of metabolic dysregulation in its pathogenesis. MASLD is defined as hepatic steatosis accompanied by at least one cardiometabolic risk factor [4]. Recent studies have shown that the global prevalence of MASLD exceeds 30%, showing an upward trend [5]. Notably, in some countries, including Türkiye, where the burden of cardiometabolic risk factors is high, the prevalence of MASLD exceeds 40% [6,7,8,9].

Although the coexistence of IBD and hepatic steatosis has been increasingly recognized, the relative contributions of metabolic and disease-related factors remain poorly defined, particularly under the updated MASLD definition. Moreover, data regarding MASLD prevalence and associated factors in patients with IBD remain limited, especially in regions with a high metabolic burden such as Türkiye. Given the high background prevalence of cardiometabolic risk factors and MASLD in Türkiye, clarifying whether hepatic steatosis in patients with IBD primarily reflects disease-specific mechanisms or the underlying regional metabolic milieu is of particular clinical relevance. Therefore, this study aimed to evaluate the prevalence of MASLD in patients with IBD in a Turkish tertiary-care cohort and to identify the metabolic and disease-related factors associated with its development.

## 2. Materials and Methods

### 2.1. Study Design and Population

This single-center, retrospective, cross-sectional study included patients with IBD who were followed at the Gastroenterology Outpatient Clinic of Umraniye Training and Research Hospital, a tertiary referral center in Türkiye, a country with a high background prevalence of cardiometabolic risk factors and MASLD.

The study protocol was approved by the Institutional Ethics Committee of Umraniye Training and Research Hospital (Decision No: 501, Date: 16 January 2025), and the study was conducted in accordance with the principles of the Declaration of Helsinki. The requirement for informed consent was waived by the Ethics Committee due to the retrospective design of the study and the use of anonymized medical records of the patients.

Adult patients (≥18 years) with a confirmed diagnosis of CD or UC who visited the outpatient clinic between September 2024 and January 2025 were retrospectively identified for inclusion. Patients were included if abdominal imaging performed within the preceding 6 months was available for assessment of hepatic steatosis. Because imaging was obtained as part of routine clinical care rather than a systematic screening protocol, the study cohort may have preferentially included patients undergoing hepatobiliary or metabolic evaluation.

### 2.2. Exclusion Criteria

Patients with any of the following conditions were excluded:

- Excessive alcohol consumption (>20 g/day for women and >30 g/day for men);
- Other chronic liver diseases (including viral hepatitis, autoimmune hepatitis, primary biliary cholangitis, primary sclerosing cholangitis, Wilson’s disease, hemochromatosis, α1-antitrypsin deficiency, or drug-induced liver injury);
- Cirrhosis or hepatocellular carcinoma;
- History of organ transplantation;
- Short bowel syndrome or patients receiving total parenteral nutrition;
- Pregnancy or lactation;
- Patients with missing biochemical, imaging, or anthropometric data for the confirmation or exclusion of MASLD.

Alcohol consumption was assessed retrospectively from routinely documented medical records, including the initial clinical history obtained at diagnosis and subsequent outpatient follow-up notes. Patients with documented alcohol consumption exceeding 20 g/day for women or 30 g/day for men were excluded from the study.

### 2.3. Definition of Inflammatory Bowel Disease

IBD diagnosis was established based on a comprehensive evaluation of clinical presentation, endoscopic findings, histopathological examination, and radiological imaging in accordance with current international guidelines. Only patients with a confirmed diagnosis of CD or UC documented in their medical records were included in the analyses.

### 2.4. Disease Location and Phenotype

The disease location in patients with CD was classified according to the Montreal classification as follows:

L1: ileal disease;
L2: colonic disease;
L3: ileocolonic disease;
L4: upper GI tract.

In patients with UC, disease extent was defined according to the Montreal classification as follows:

E1: ulcerative proctitis;
E2: left-sided colitis;
E3: extensive colitis/pancolitis.

A history of IBD-related intestinal surgery and the presence of fistulas, abscesses, or strictures were also recorded.

### 2.5. Assessment of Disease Activity

Disease activity was assessed using validated disease-specific activity scores at the time of clinical assessment closest to the index visit. In patients with CD, disease activity was assessed using the Crohn’s Disease Activity Index (CDAI). Patients with a CDAI score < 150 were considered to be in clinical remission, whereas those with a CDAI score ≥ 150 were classified as having an active disease. In patients with UC, disease activity was assessed using the Disease Activity Index (DAI). Patients with a DAI score < 2 were considered to be in clinical remission, while those with a DAI score ≥ 2 were classified as having an active disease. Disease activity score data were unavailable for a small number of patients (9) because of incomplete documentation in the retrospective medical records. Disease activity status (active disease vs. remission) was included as a categorical variable in the subsequent analyses.

### 2.6. Definition of MASLD

MASLD was diagnosed according to the 2023 Delphi Consensus criteria [4]. Accordingly, in patients with radiologically confirmed hepatic steatosis, the presence of at least one of the following five cardiometabolic risk factors (CMRF) was required for a MASLD diagnosis:

- BMI ≥ 25 kg/m^2^, or waist circumference > 94 cm in men; >80 cm in women;
- Fasting blood glucose ≥ 100 mg/dL or established diagnosis of T2D;
- Blood pressure ≥ 130/85 mmHg or the use of antihypertensive medication;
- Plasma triglycerides ≥ 150 mg/dL or the use of lipid-lowering medication;
- Plasma HDL cholesterol < 40 mg/dL (men) or <50 mg/dL (women); or the use of lipid-lowering medication.

### 2.7. Assessment of Hepatic Steatosis

Hepatic steatosis was diagnosed using abdominal ultrasonography (USG) in 163 patients (84%), computed tomography (CT) in 16 (8.2%), and magnetic resonance imaging (MRI) in 15 (7.7%) based on the review of medical records. Steatosis was determined based on the formal radiology reports available in the electronic medical record. Images were not re-reviewed for the purposes of this study. Hepatic steatosis was assessed qualitatively on ultrasonography based on increased hepatic echogenicity relative to renal cortex, on CT based on decreased liver attenuation relative to the spleen, and on MRI based on signal loss of the hepatic parenchyma on opposed-phase images compared with in-phase images. No quantitative assessment methods, such as controlled attenuation parameter (CAP), transient elastography or MRI-based fat quantification, were systematically available and therefore were not included in the analysis.

### 2.8. Data Collection

Demographic data (age and sex), anthropometric measurements (height, body weight, body mass index [BMI], and waist circumference), IBD-related characteristics (disease duration, location, and activity; history of IBD-related surgery), medical therapies (corticosteroids, immunomodulators, and biologic agents), and imaging reports were retrospectively obtained from medical records.

Laboratory data included fasting plasma glucose, lipid profile, liver enzymes, complete blood count, serum albumin, and C-reactive protein (CRP) levels.

### 2.9. Statistical Analysis

Statistical analyses were performed using IBM SPSS Statistics (version 27; IBM Corp., Armonk, NY, USA). The Kolmogorov–Smirnov test was used to assess the distribution. Continuous variables were expressed as mean ± standard deviation (SD) for normally distributed data and as median (interquartile range [IQR]) for non-normally distributed data. Categorical variables were presented as numbers and percentages.

Comparisons between the MASLD and non-MASLD groups were performed using Student’s *t*-test or Mann–Whitney U test for continuous variables and the Pearson’s chi-square test or Fisher’s exact test for categorical variables. A *p* value < 0.05 was considered statistically significant.

Variables with a *p* value < 0.10 in the univariable analyses were considered candidates for multivariable logistic regression analysis. A forward conditional logistic regression procedure was used for variable selection. Multicollinearity among candidate variables was assessed using variance inflation factors (VIFs).

## 3. Results

### 3.1. Study Population and Baseline Characteristics

**Figure 1:**
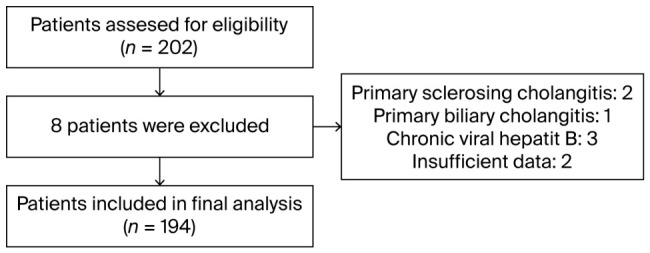
Flowchart of patient selection.

A total of 202 patients with inflammatory bowel disease were screened. Eight patients were excluded due to concomitant liver diseases or insufficient data, leaving 194 patients for the final analysis (Figure 1). Among the patients included, 119 (61.3%) were diagnosed with CD and 75 (38.7%) with UC. The mean age of the study population was 43.7 ± 13.3 years, and 117 patients (60.3%) were male. The baseline demographic, clinical, and laboratory characteristics of the patients were presented in Table 1.

Among patients with a history of IBD-related surgery (*n* = 61, 31.4%), the most common surgical procedure was ileocecal resection (30, 49.2%), followed by small bowel resection (14, 23%). Total proctocolectomy was performed in seven patients (11.5%), diversion surgery in eight (13.1%), and segmental colonic resection in two patients (3.3%).

**Figure 2:**
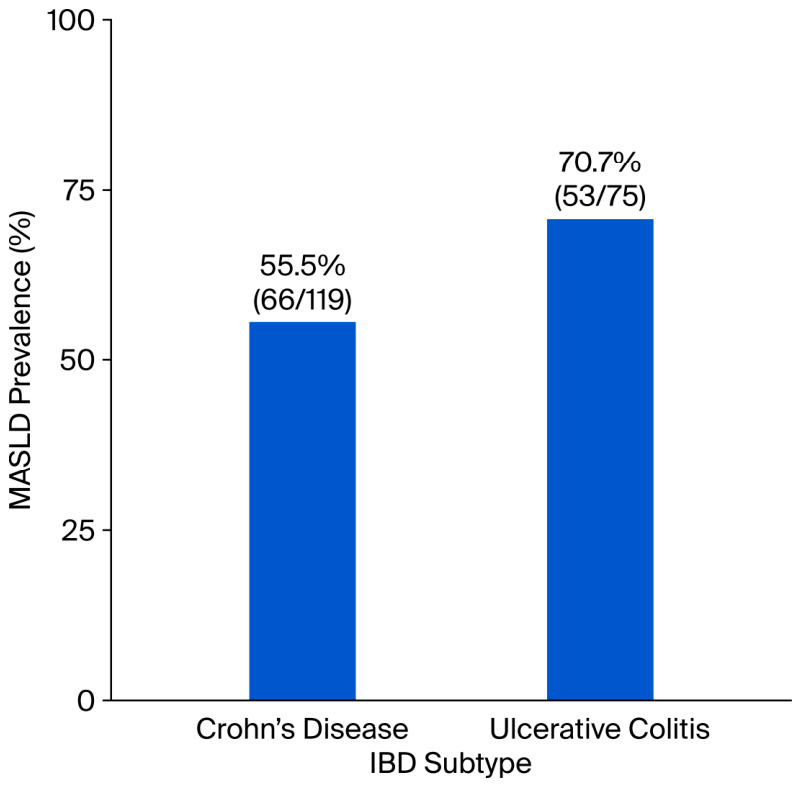
Prevalence of MASLD according to IBD subtype.

Overall, MASLD was identified in 119 patients, yielding a prevalence rate of 61.3%. As shown in Table 2, the prevalence of MASLD was significantly higher in patients with UC than in those with CD (70.7% vs. 55.5%; *p* = 0.036) (Figure 2). At least one CMRF was present in 86.1% of the study population, while 49.5% had at least two and 26.3% had at least three CMRF.

### 3.2. Comparison of Clinical Characteristics

The clinical and disease-related characteristics of the patients according to MASLD status are shown in Table 2. Patients with MASLD were significantly older than those without MASLD (median age: 48 [IQR: 40–57] vs. 34 [IQR: 28–45] years; *p* < 0.001). Male sex was more frequent in the MASLD group; however, this difference did not reach statistical significance (64.7% vs. 53.3%, *p* = 0.133).

The anthropometric measurements differed significantly between the groups. Patients with MASLD had significantly higher BMI and waist circumference than those in the non-MASLD group (both *p* < 0.001). Obesity (BMI ≥ 30 kg/m^2^) was present in 38.7% of patients with MASLD and 16% of those without MASLD (*p* < 0.001).

Regarding IBD-related characteristics, disease duration did not differ significantly between the MASLD and non-MASLD groups (*p* = 0.219). However, UC diagnosis was more frequent among patients with MASLD than among those without MASLD (44.5% vs. 29.3%; *p* = 0.036). Additional analyses of IBD-specific disease characteristics showed no significant association between MASLD and disease extent in patients with ulcerative colitis (*p* = 0.213) or disease location in patients with Crohn’s disease (*p* = 0.171).

The disease activity scores differed between the groups. Patients with MASLD had significantly lower CDAI scores compared with non-MASLD patients (21.5 [IQR: 9.2–42] vs. 46 [IQR: 19–102]; *p* = 0.002). Similarly, in patients with UC, DAI scores were lower in the MASLD group (0 [IQR: 0–1] vs. 1.5 [IQR: 0–3]; *p* < 0.001). When categorized, clinical remission was more frequent in the MASLD group than in the non-MASLD group (79.8% vs. 69.3%; *p* = 0.044). A history of IBD-related surgery was significantly less frequent in the MASLD group (23.5% vs. 44%; *p* = 0.003).

With respect to medical therapy, the use of biologic agents was significantly lower in patients with MASLD compared with non-MASLD patients (54.6% vs. 77.3%; *p* = 0.001). The use of corticosteroids, immunomodulators, and mesalamine did not differ significantly between the groups.

Hypertension was significantly more prevalent in the MASLD group than in the non-MASLD group (23.5% vs. 12%, *p* = 0.047). Diabetes mellitus was more frequent among patients with MASLD, showing a borderline statistical association (10.9% vs. 2.7%; *p* = 0.051). Hyperlipidemia and smoking status did not differ significantly between the groups.

The distribution of imaging modalities differed significantly between patients with and without MASLD (*p* < 0.001). Among patients with MASLD, hepatic steatosis assessment was based on ultrasonography (USG) in 93.3%, CT in 2.5%, and MRI in 4.2%, whereas in the non-MASLD group the corresponding proportions were 69.3%, 17.3%, and 13.3%, respectively. When analyzed according to imaging modality, MASLD was identified in 68.1% (111/163) of patients assessed by USG, compared with 33.3% (5/15) of those assessed by MRI and 18.8% (3/16) of those assessed by CT.

### 3.3. Comparison of Laboratory Parameters

The laboratory parameters according to the MASLD status are summarized in Table 3. Patients with MASLD exhibited significantly higher fasting plasma glucose levels compared with non-MASLD patients (95 [IQR: 87–106] vs. 88 [IQR: 80–94] mg/dL; *p* < 0.001). Lipid parameters were significantly less favorable in patients with MASLD. Total cholesterol, triglyceride, and low-density lipoprotein (LDL) cholesterol levels were significantly higher in the MASLD group, whereas high-density lipoprotein (HDL) cholesterol levels were significantly lower (all *p* < 0.05). Hemoglobin levels were modestly but significantly higher in patients with MASLD (*p* = 0.006), whereas platelet counts and CRP levels did not differ significantly between the groups.

### 3.4. Logistic Regression Analysis

In univariable logistic regression analyses, older age, higher BMI, increased waist circumference, UC diagnosis, disease activity (remission), presence of fistula, absence of prior surgery, lower biologic agent use, high fasting glucose, and high lipid parameters were significantly associated with MASLD (Table 4).

In the first model of multivariable logistic regression analysis, which included only IBD-related variables and age, older age (OR 1.070, 95% CI: 1.041–1.099; *p* < 0.001) was associated with higher odds of MASLD, whereas prior IBD-related surgery was inversely associated with MASLD (OR 0.495, 95% CI: 0.251–0.973; *p* = 0.042).

**Figure 3:**
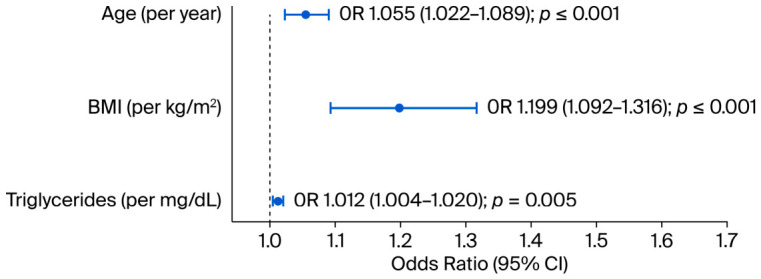
Forest plot showing independent factors associated with MASLD in multivariable logistic regression analysis.

In the second model, which additionally included metabolic parameters and other relevant covariates identified in the univariable analyses, only age (OR 1.055, 95% CI: 1.022–1.089; *p* < 0.001), BMI (OR 1.199, 95% CI: 1.092–1.316; *p* < 0.001), and triglyceride levels (OR 1.012, 95% CI: 1.004–1.020; *p* = 0.005) remained independently associated with MASLD. The association between prior surgery and MASLD was no longer statistically significant after adjusting for metabolic parameters. The results of the multivariable logistic regression analyses are shown in Table 5. The magnitude and direction of these associations are illustrated in the forest plot shown in Figure 3.

As a sensitivity analysis, the multivariable logistic regression model was repeated in the subgroup of patients assessed by ultrasonography only (*n* = 163). The results were broadly consistent with the primary analysis. Age and triglyceride levels remained independently associated with MASLD, whereas waist circumference replaced BMI as the anthropometric measure independently associated with MASLD (Supplementary Table S1). Additionally, sex was added to the final multivariable logistic regression model. Male sex was not independently associated with MASLD (OR 1.42, 95% CI 0.65–3.09, *p* = 0.380), and the associations of age, BMI, and triglyceride levels with MASLD remained essentially unchanged.

## 4. Discussion

In this cross-sectional study, MASLD was identified in 61.3% of selected patients with IBD who had abdominal imaging available within the previous 6 months, according to the 2023 Delphi Consensus criteria, indicating a substantial burden of hepatic steatosis with metabolic dysfunction in this population. Although MASLD was more frequent in patients with UC than in those with CD, multivariable analyses demonstrated that age, BMI, and triglyceride levels, rather than IBD-related disease characteristics, were independently associated with MASLD. These findings suggest that MASLD in patients with IBD largely mirrors the underlying cardiometabolic risk profile of the background population rather than representing a predominantly disease-specific hepatic manifestation.

This issue may be particularly relevant in countries such as Türkiye, where the prevalence of cardiometabolic risk factors and MASLD is substantially higher than the global average. In this context, the high MASLD prevalence observed in our cohort likely reflects the regional metabolic burden in addition to IBD-related factors. Given that a large proportion of our cohort had at least one cardiometabolic risk factor, our findings support the concept that metabolic dysfunction is closely linked to MASLD in most patients with IBD.

The reported prevalence of hepatic steatosis in patients with IBD varies considerably across studies, largely depending on the diagnostic modality and disease definition used. Earlier studies based on the NAFLD definition reported prevalence rates ranging from approximately 24% to 34%, consistently higher than those observed in the general population [10,11,12,13]. More recent studies using the updated MASLD criteria have demonstrated prevalence rates between 18% and 44% in IBD cohorts [14,15,16,17,18]. In parallel, epidemiological data from Türkiye and the broader Middle East and North Africa (MENA) region indicate a particularly high burden of MASLD in the general population, especially among individuals with cardiometabolic risk factors [6,7,8,9,19,20,21].

The key finding of this study was that MASLD in patients with IBD appears to be more strongly associated with metabolic risk factors rather than disease-related characteristics. In the initial multivariable model, which included only IBD-related variables, older age was associated with increased odds of MASLD, whereas prior IBD-related surgery showed an inverse association with MASLD. However, after adjusting for metabolic parameters, only age, BMI, and triglyceride levels remained independently associated with MASLD, whereas IBD-related variables lost statistical significance. These findings suggest that the associations between IBD-related factors and MASLD are largely mediated through their impact on patients’ metabolic profiles rather than reflecting a direct effect, consistent with previous studies highlighting the main role of metabolic dysfunction in hepatic steatosis among IBD populations [12,13,18,22,23,24,25].

Interpretation of the relationship between IBD-related factors and MASLD remains challenging because disease severity, treatment exposure, nutritional status, and surgical history are closely interrelated and may influence metabolic risk profiles in different ways. Patients with more severe or longstanding disease may have lower BMI because of malnutrition or prior intestinal resection, whereas prolonged corticosteroid exposure may contribute to insulin resistance, central adiposity, and dyslipidemia. Conversely, effective disease control may reduce systemic inflammation and mitigate metabolic risk over time. In addition, non-metabolic mechanisms, including alterations in the gut–liver axis, have also been implicated in hepatic steatosis in IBD. These overlapping effects make it difficult to determine the independent contribution of IBD-related factors to MASLD risk, particularly in cross-sectional studies [26].

The relationship between corticosteroid use, biologic therapy, surgical history, and MASLD remains incompletely understood. Although biologic agents have been proposed to reduce hepatic steatosis by controlling systemic inflammation and disease activity, current evidence does not support this assumption [25,27]. Similarly, while some studies have suggested that intestinal resection may predispose patients to hepatic steatosis, subsequent research has failed to confirm surgical history as an independent risk factor in multivariate analyses [18,25]. In our study, the use of biologic therapy and history of IBD-related surgery were less frequent among patients with MASLD; however, these associations were not significant after adjusting for metabolic factors. This suggests that their apparent effects are likely indirect and mediated by differences in metabolic profiles, such as a lower BMI, rather than reflecting a direct role. On the other hand, corticosteroid use was not associated with MASLD in our cohort. Previous studies have not consistently identified steroid use as an independent risk factor for MASLD in patients with IBD [25,28]. However, some reports suggest that cumulative corticosteroid exposure may contribute to the development of hepatic steatosis [29]. Corticosteroid use was assessed as a binary variable; the lack of data on cumulative doses and the exact duration of therapy may have limited our ability to identify a dose-dependent relationship.

The distribution of MASLD across IBD subtypes also remains controversial in the literature. While some studies have identified CD as a significant risk factor, reporting a higher prevalence of NAFLD in this subgroup, others have observed a greater frequency in patients with UC [26]. Notably, one study emphasized that the higher MASLD prevalence in UC may be driven by metabolic factors (BMI, frequent corticosteroid use) rather than being a direct consequence of disease activity [30]. In our cohort, patients with UC were generally older and had higher BMI values, which are established risk factors for MASLD. In contrast, patients with CD more frequently had a history of surgery and biologic agent use, which are associated with lower body weight and altered nutritional status. Importantly, UC diagnosis was not an independent predictor of MASLD in multivariable analysis. The significant association observed between MASLD and UC in the univariable analysis likely reflects the older age and less favorable metabolic profile (including higher BMI) of the UC subgroup rather than a UC-specific effect. Because MASLD is typically a long-standing and often silent metabolic condition, metabolic abnormalities may have preceded the diagnosis of IBD, particularly in older patients. Given the cross-sectional design of our study, temporal and causal relationships cannot be established. Therefore, the apparent association between IBD subtype and MASLD should be interpreted cautiously, acknowledging that differences in age and metabolic profile may account for much of the observed association.

In our cross-sectional analysis, disease activity scores were lower, and clinical remission was more frequent in patients with MASLD; however, disease activity was not identified as an independent risk factor in the multivariable analysis. It is important to note that disease activity scores reflect inflammatory status at a single time point and may not adequately represent the cumulative inflammatory burden over the disease course. Therefore, the lack of an independent association should be interpreted cautiously and does not necessarily indicate the absence of a relationship. Some previous studies have suggested that frequent relapses and high disease activity scores may be associated with an increased risk of hepatic steatosis [27,29]. However, a growing body of evidence has failed to establish a consistent correlation, and many studies have reported no such association. Furthermore, recent research paradoxically suggests that clinical remission and lower disease activity scores may be more closely linked to steatosis [26]. This phenomenon may be mediated by the positive impact of clinical stability on nutritional status and subsequent increases in BMI, which may ultimately predispose patients to metabolic dysfunction. Taken together, these findings indicate that the relationship between inflammatory activity and MASLD remains complex.

While our study focused primarily on standard clinical features such as disease activity, surgical history, and biologic therapies, these variables may not fully capture the complex intestinal mechanisms underlying the gut–liver axis in patients with IBD. Growing evidence suggests that gut dysbiosis-induced reductions in short-chain fatty acid production and alterations in bile acid metabolism, together with the accumulation of dysbiotic microbial metabolites, may contribute to both local and systemic inflammation. In addition, intestinal barrier dysfunction may facilitate the translocation of endotoxins, such as lipopolysaccharides, into the portal and systemic circulation, thereby contributing to hepatic inflammation, fibrosis, and metabolic dysfunction associated with MASLD [31]. Recent experimental studies have further highlighted these mechanistic links. For instance, alterations in the gut microbiota have been shown to directly regulate lipid droplet dynamics in fatty liver models [32], while modulation of chronic intestinal inflammation through specific oligosaccharides has been associated with favorable changes in gut microbial composition [33]. These findings suggest that intestinal microenvironmental alterations may contribute to MASLD development in patients with IBD beyond conventional metabolic risk factors. Future prospective studies incorporating fecal microbiota profiling, fecal metabolomics, bile acid profiling, fecal calprotectin, and markers of intestinal permeability are warranted to better elucidate these pathophysiological pathways.

From a clinical perspective, MASLD is highly prevalent among patients with IBD and is strongly associated with metabolic risk factors, similar to the trends observed in the general population. Notably, MASLD was identified in 22.6% of lean patients (14/62), indicating that hepatic steatosis may develop even in the absence of obesity, a phenomenon consistent with lean MASLD. This prevalence is in line with a recent report describing MASLD in lean individuals [34]. These findings underscore the importance of routine metabolic risk assessment and proactive screening for MASLD as part of comprehensive IBD management, regardless of disease activity or treatment status. Routine assessment of metabolic risk factors and consideration of liver imaging in appropriate patients may facilitate earlier identification of MASLD. Non-invasive fibrosis assessment tools, such as FIB-4 and elastography, may be useful for risk stratification in selected patients.

This study had several limitations. Its retrospective and cross-sectional design precludes causal inference. The diagnosis of hepatic steatosis relied on heterogeneous imaging modalities (USG, CT, and MRI) rather than liver biopsy, which is not feasible for screening. Although these are standard clinical tools, their varying sensitivities and specificities for detecting low-grade steatosis may have introduced diagnostic variability. In addition, fibrosis severity was not systematically assessed, limiting evaluation of the prognostic implications of MASLD in this cohort. Additionally, reliance on available imaging records may have resulted in selection bias. Because imaging was performed as part of routine clinical care rather than systematic screening, MASLD prevalence may have been overestimated. Therefore, the reported prevalence should be understood as reflecting the subset of IBD patients who underwent imaging, rather than representing the prevalence in the overall IBD population treated in a tertiary care setting. Furthermore, several metabolic variables evaluated as potential predictors are components of or closely related to the cardiometabolic criteria used in the MASLD definition. Accordingly, these associations should be interpreted as reflecting the cardiometabolic profile incorporated into the MASLD definition rather than as completely independent predictors. The predominance of patients in clinical remission may limit the evaluation of the direct impact of active inflammation on these results. Finally, as a single-center study, our findings reflect the specific metabolic profile of our regional cohort and may not be fully generalizable to other populations with different metabolic backgrounds. Despite these limitations, the use of the updated MASLD definition and comprehensive evaluation of metabolic risk factors in a relatively large IBD cohort represent the key strengths of this study.

## 5. Conclusions

MASLD prevalence was high among selected IBD patients with available abdominal imaging and appears to be more strongly associated with metabolic risk factors rather than disease-specific characteristics, largely reflecting the underlying regional cardiometabolic burden. These findings highlight the importance of routine metabolic risk assessment and proactive MASLD screening as part of comprehensive long-term IBD care. Prospective longitudinal studies incorporating fibrosis assessment are needed to better clarify the causal relationship between metabolic dysfunction, IBD-related factors, and MASLD progression.

## Figures and Tables

**Table 1:** Baseline Characteristics and Laboratory Parameters of the Study Population.

	Crohn’s Disease (*n* = 119)	Ulcerative Colitis (*n* = 75)	Total (*n* = 194)
Age (years)	40.3 ± 12.6	49 ± 12.5	43.7 ± 13.3
Sex, *n* (%)			
Female	50 (42%)	27 (36%)	77 (39.7%)
Male	69 (58%)	48 (64%)	117 (60.3%)
BMI (kg/m^2^)	27.5 ± 5.4	27.6 ± 4.8	27.5 ± 5.1
Waist circumference (cm)	95.7 ± 13.1	95.5 ± 12.5	95.6 ± 12.8
Disease Duration (month)	96 (58–152)	94 (48–147)	95 (52–148)
Disease Location, *n* (%)	L1: 46 (38.7%) L2: 69 (58%) L3: 4 (3.4%)	E1: 26 (34.7%) E2: 24 (32%) E3: 25 (33.3%)	-
Disease Activity *			
Active disease, *n* (%)	22 (18.5%)	22 (29.3%)	44 (22.7%)
Remission, *n* (%)	97 (81.5%)	50 (66.7%)	147 (75.8%)
Disease Activity Scores **			
CDAI (*n* = 119)	57.4 ± 76.3	-	-
DAI (*n* = 66)	-	0.9 ± 1.6	-
Complications			
Abscess, *n* (%)	24 (20.2%)	1 (1.3%)	25 (12.9%)
Fistula, *n* (%)	39 (32.8%)	0 (0%)	39 (20.1%)
Stricture, *n* (%)	49 (41.2%)	0 (0%)	49 (25.3%)
Prior Surgery, *n* (%)	55 (46.2%)	6 (8%)	61 (31.4%)
Mesalamine, *n* (%)	98 (82.4%)	75 (100%)	173 (89.2%)
Corticosteroids, *n* (%)	76 (63.9%)	41 (54.7%)	117 (60.3%)
Immunomodulators, *n* (%)	113 (95%)	43 (57.3%)	156 (80.4%)
Biologic Agent (ongoing), *n* (%)	100 (84%)	23 (30.7%)	123 (63.4%)
Diabetes Mellitus, *n* (%)	9 (7.6%)	6 (8%)	15 (7.7%)
Hypertension, *n* (%)	17 (14.3%)	20 (26%)	37 (19.1%)
Hyperlipidemia, *n* (%)	7 (5.9%)	7 (9.3%)	14 (7.2%)
Active Smoking, *n* (%)	40 (33.6%)	14 (18.7%)	54 (27.8%)
CMRF ≥ 1, *n* (%)	101 (84.9%)	66 (88%)	167 (86.1%)
CMRF ≥ 2, *n* (%)	56 (47.1%)	40 (53.3%)	96 (49.5%)
CMRF ≥ 3, *n* (%)	27 (22.7%)	24 (32%)	51 (26.3%)
MASLD, *n* (%)	66 (55.5%)	53 (70.7%)	119 (61.3%)
Fasting Glucose (mg/dL)	90 (83–103)	94 (88–105)	92 (85–104)
AST (IU/L)	18 (15–21)	18 (14–22)	18 (15–21)
ALT (IU/L)	17 (11–23)	17 (12–24)	17 (11–23)
GGT (IU/L)	19 (12–30)	20 (14–30)	19 (13–30)
Total cholesterol (mg/dL)	172 (149–194)	195 (149–223)	178 (149–212)
Triglycerides (mg/dL)	127 (89–161)	105.5 (78–147)	117 (84–155)
LDL-Cholesterol (mg/dL)	94 (73–118)	120 (82–143)	101 (79–128)
HDL-Cholesterol (mg/dL)	46 (40–57)	50 (42–57)	48 (40–57)
Hemoglobin (g/dL)	13.7 ± 2	14.2 ± 1.5	13.9 ± 1.8
Platelet Count (×10^3^/uL)	274 (228–329)	255 (219–309)	266 (222–319)
CRP (mg/L)	2.6 (1–6.6)	1.5 (0.7–3.5)	2 (0.8–5)

Data are presented as *n* (%), mean ± SD, or median (IQR). Abbreviations: BMI, body mass index; CDAI, Crohn’s disease activity index; DAI, disease activity index; CMRF, cardiometabolic risk factors; MASLD, metabolic dysfunction-associated steatotic liver disease; AST, aspartate aminotransferase; ALT, alanine aminotransferase; GGT, gamma-glutamyl transferase; LDL, low-density lipoprotein; HDL, high-density lipoprotein; CRP, c-reactive protein; SD, standard deviation; IQR, interquartile range. * Disease activity data were available for 191 patients. ** Disease activity score data were available in 119 CD and 66 UC patients.

**Table 2:** Comparison of the Baseline Characteristics According to the MASLD Status.

Parameter	MASLD (*n* = 119)	Non-MASLD (*n* = 75)	*p* Value
Age (years)	48 (40–57)	34 (28–45)	<0.001
Sex, *n* (%)			
Female	42 (35.3%)	35 (46.7%)	0.133
Male	77 (64.7%)	40 (53.3%)	
BMI (kg/m^2^)	28 (25.9–32.2)	24 (21–26.8)	<0.001
BMI Categories, *n* (%)			
<25	14 (11.8%)	48 (64%)	
25–29.9	59 (49.6%)	15 (20%)	<0.001
≥30	46 (38.7%)	12 (16%)	
Waist circumference (cm)	99 (92–107)	87 (79.2–97.7)	<0.001
IBD subtype, *n* (%)			
CD	66 (55.5%)	53 (70.7%)	0.036
UC	53 (44.5%)	22 (29.3%)	
Disease Duration (month)	100 (61–147)	89 (43–156)	0.219
Disease Activity *			
Active disease, *n* (%)	21 (17.6%)	23 (30.7%)	0.044
Remission, *n* (%)	95 (79.8%)	52 (69.3%)	
Disease Activity Scores **			
CDAI	21.5 (9.2–42)	46 (19–102)	0.002
DAI	0 (0–1)	1.5 (0–3)	<0.001
Complications			
Abscess, *n* (%)	12 (10.1%)	13 (17.3%)	0.142
Fistula, *n* (%)	18 (15.1%)	21 (28%)	0.029
Stricture, *n* (%)	26 (21.8%)	23 (30.7%)	0.179
Prior Surgery, *n* (%)	28 (23.5%)	33 (44%)	0.003
Mesalamine, *n* (%)	107 (89.9%)	66 (88%)	0.676
Corticosteroids, *n* (%)	70 (58.8%)	47 (62.7%)	0.594
Immunomodulators, *n* (%)	94 (79%)	62 (82.7%)	0.530
Biologic use (ongoing), *n* (%)	65 (54.6%)	58 (77.3%)	0.001
Diabetes Mellitus, *n* (%)	13 (10.9%)	2 (2.7%)	0.051
Hypertension, *n* (%)	28 (23.5%)	9 (12%)	0.047
Hyperlipidemia, *n* (%)	9 (7.6%)	9 (6.7%)	0.814
Active Smoking, *n* (%)	37 (31.1%)	17 (22.7%)	0.202
CMRF ≥ 2, *n* (%)	78 (65.5%)	18 (24%)	<0.001
CMRF ≥ 3, *n* (%)	41 (34.5%)	10 (13.3%)	0.003

Data are presented as *n* (%), mean ± SD, or median (IQR). Categorical variables were analyzed using Pearson’s chi-square test or Fisher’s exact test. Continuous variables were analyzed using an unpaired *t*-test if normally distributed or Mann–Whitney test if skewed. *p* < 0.05, statistically significant. Abbreviations: MASLD, metabolic dysfunction-associated steatotic liver disease; BMI, body mass index; CD, Crohn’s disease; UC, ulcerative colitis; CDAI, Crohn’s disease activity index; DAI, disease activity index; CMRF, cardiometabolic risk factors; SD, standard deviation; IQR, interquartile range. * Disease activity data were available for 191 patients. ** Disease activity score data were available in 119 CD and 66 UC patients.

**Table 3:** Comparison of Laboratory Parameters according to MASLD Status.

Parameter	MASLD (*n* = 119)	Non-MASLD (*n* = 75)	*p* Value
Glucose (Fasting) (mg/dL)	95 (87–106)	88 (80–94)	<0.001
AST (IU/L)	18 (15–22)	16 (14–21)	0.129
ALT (IU/L)	19 (12–26)	15 (10–21)	0.008
GGT (IU/L)	22 (15–32)	16 (11–23)	<0.001
Total cholesterol (mg/dL)	189 (163–216)	156.5 (138.5–185)	<0.001
Triglycerides (mg/dL)	132 (99–172)	93 (64–129)	<0.001
LDL-Cholesterol (mg/dL)	115 (86–140)	86 (65–112)	<0.001
HDL-Cholesterol (mg/dL)	46 (39–54)	50.5 (42–61.5)	0.021
Hemoglobin (g/dL)	14.4 (13.3–15.4)	13.4 (12.2–15.2)	0.006
Platelet Count (×10^3^/uL)	269 (224–309)	262 (219–338)	0.632
CRP (mg/L)	2.1 (1–4.8)	1.7 (0.7–6.1)	0.438

Data are presented as *n* (%), mean ± SD, or median (IQR). Continuous variables were analyzed with an unpaired *t*-test if normally distributed or Mann–Whitney test if skewed. *p* < 0.05, statistically significant. Abbreviations: MASLD, metabolic dysfunction-associated steatotic liver disease; AST, aspartate aminotransferase; ALT, alanine aminotransferase; GGT, gamma-glutamyl transferase; LDL, low-density lipoprotein; HDL, high-density lipoprotein; CRP, c-reactive protein; SD, standard deviation; IQR, interquartile range.

**Table 4:** Univariable Logistic Regression Analysis of MASLD Risk Factors.

Parameter	Univariable Analysis	
	OR (95% CI)	*p* Value
Age (years)	1.075 (1.045–1.105)	<0.001
BMI (kg/m^2^)	1.283 (1.177–1.400)	<0.001
Waist circumference (cm)	1.096 (1.060–1.134)	<0.001
Diagnosis (UC)	1.935 (1.046–3.577)	0.035
Disease Activity (Active disease)	0.500 (0.253–0.988)	0.046
Fistula	0.458 (0.225–0.933)	0.031
Prior Surgery	0.392 (0.210–0.730)	0.003
Biologic Agent (ongoing)	0.353 (0.184–0.676)	0.002
Diabetes Mellitus	4.476 (0.981–20.431)	0.053
Hypertension	2.256 (0.999–5.098)	0.050
CMRF ≥ 2	5.417 (2.800–10.480)	<0.001
CMRF ≥ 3	3.088 (1.429–6.675)	0.004
Glucose (Fasting) (mg/dL)	1.048 (1.022–1.074)	<0.001
ALT (IU/L)	1.032 (1.004–1.061)	0.027
GGT (IU/L)	1.035 (1.010–1.062)	0.007
Total cholesterol (mg/dL)	1.016 (1.008–1.025)	<0.001
Triglycerides (mg/dL)	1.015 (1.008–1.022)	<0.001
LDL-Cholesterol (mg/dL)	1.017 (1.007–1.026)	<0.001
HDL-Cholesterol (mg/dL)	0.979 (0.958–1.000)	0.055
Hemoglobin (g/dL)	1.248 (1.061–1.468)	0.007

*p* < 0.05, statistically significant. Abbreviations: MASLD, metabolic dysfunction-associated steatotic liver disease; OR, odds ratio; BMI, body mass index; UC, ulcerative colitis; CMRF, cardiometabolic risk factors; ALT, alanine aminotransferase; GGT, gamma-glutamyl transferase; LDL, low-density lipoprotein; HDL, high-density lipoprotein.

**Table 5:** Multivariable Logistic Regression Analysis of MASLD Risk Factors.

Parameter	Multivariable Analysis	
	OR (95% CI)	*p* Value
Model 1		
Age (years)	1.070 (1.041–1.099)	<0.001
Prior Surgery	0.495 (0.251–0.973)	0.042
Model 2		
Age (years)	1.055 (1.022–1.089)	<0.001
BMI (kg/m^2^)	1.199 (1.092–1.316)	<0.001
Triglycerides (mg/dL)	1.012 (1.004–1.020)	0.005

*p* < 0.05, statistically significant. Abbreviations: MASLD, metabolic dysfunction-associated steatotic liver disease; OR, Odds ratio; BMI, body mass index. Model 1 included only IBD-related variables (age, diagnosis, disease activity, presence of fistula, prior IBD-related surgery, and ongoing use of biologic agents) that showed a *p* value < 0.10 in univariable analyses to assess IBD-specific associations with MASLD. Model 2 additionally included all metabolic parameters and other relevant covariates that showed a *p* value < 0.10 in univariable analyses, allowing the evaluation of whether the IBD-related associations were attenuated after adjustment for metabolic risk factors.

## Data Availability

The data that support the findings of this study are available from the corresponding author, H.A., upon reasonable request.

## Supplementary Materials

The following supporting information can be downloaded at https://www.mdpi.com/article/10.3390/metabo16070450/s1, Table S1: Sensitivity analysis restricted to patients evaluated by ultrasonography: multivariable logistic regression analysis of factors independently associated with MASLD (*n* = 163).

## Abbreviations

The following abbreviations are used in this manuscript:

ALT	Alanine aminotransferase
AST	Aspartate aminotransferase
BMI	Body mass index
CAP	Controlled attenuation parameter
CD	Crohn’s disease
CDAI	Crohn’s disease activity index
CI	Confidence interval
CMRF	Cardiometabolic risk factors
CRP	C-reactive protein
CT	Computed tomography
DAI	Disease activity index
EIM	Extraintestinal manifestations
GGT	Gamma-glutamyl transferase
HDL	High-density lipoprotein
IBD	Inflammatory bowel disease
IQR	Interquartile range
LDL	Low-density lipoprotein
MASLD	Metabolic dysfunction-associated steatotic liver disease
MENA	Middle East and North Africa
MRI	Magnetic resonance imaging
NAFLD	Non-alcoholic fatty liver disease
OR	Odds ratio
SD	Standard deviation
T2D	Type 2 diabetes
UC	Ulcerative colitis
USG	Ultrasonography
