# Supplementary material for: Metabolic Dysfunction-Associated Steatotic Liver Disease in Inflammatory Bowel Disease: A Cross-Sectional Study of Prevalence and Associated Factors
Source: Metabolites. 2026 Jun 26;16(7):450. doi: 10.3390/metabo16070450 (PMC13414004; doi:10.3390/metabo16070450)
Supplement: Supplementary file 1 [file metabolites-16-00450-s001.zip › metabolites-4357724-supplementary.pdf]

Supplementary Table S1. Sensitivity analysis restricted to patients evaluated by ultrasonography: multivariable logistic regression analysis of factors independently associated with MASLD (n = 163).

Parameter	Multivariable Analysis	
	**OR (95% CI)**	***p* Value**
Age (years)	1.050 (1.013-1.088)	0.007
Waist circumference (cm)	1.087 (1.040-1.135)	<0.001
Triglycerides (mg/dL)	1.012 (1.003-1.021)	0.008

$p < 0.05$, statistically significant. Abbreviations: MASLD, metabolic dysfunction-associated steatotic liver disease; OR, Odds ratio. All metabolic parameters and other relevant covariates that showed a p value <0.10 in univariable analyses included in the final analyses.
